# Addendum: Yang, Z., *et al*. Multi-Toxic Endpoints of the Foodborne Mycotoxins in Nematode *Caenorhabditis elegans*. *Toxins (Basel)*, 2015, *7*(12), 5224–5235

**DOI:** 10.3390/toxins8050141

**Published:** 2016-05-05

**Authors:** Zhendong Yang, Kathy S. Xue, Xiulan Sun, Lili Tang, Jia-Sheng Wang

**Affiliations:** 1 Synergetic Innovation Center of Food Safety and Nutrition, School of Food Science, Jiangnan University, Wuxi, Jiangsu 214122, China; zdyang777@163.com (Z.Y.); sxlzzz@jiangnan.edu.cn (X.S.); 2 Department of Environmental Health Science, University of Georgia, Athens, GA 30602, USA; ksxue@uga.edu (K.S.X.); jswang@uga.edu (J.-S.W.)

The authors wish to add the first affiliation address “Synergetic Innovation Center of Food Safety and Nutrition, School of Food Science, Jiangnan University” to the correspondence author, Lili Tang’s affiliations on the first page of their paper published in *Toxins* [1]. The authors apologize for any inconvenience this may cause.

For any questions, please contact the editorial office of the journal or support@mdpi.com.
